# Prognostic Value and Outcome for ETV6/RUNX1-Positive Pediatric Acute Lymphoblastic Leukemia: A Report From the South China Children’s Leukemia Group

**DOI:** 10.3389/fonc.2021.797194

**Published:** 2021-12-20

**Authors:** Kun-yin Qiu, Hong-gui Xu, Xue-qun Luo, Hui-rong Mai, Ning Liao, Li-hua Yang, Min-cui Zheng, Wu-qing Wan, Xue-dong Wu, Ri-yang Liu, Qi-wen Chen, Hui-qin Chen, Xiao-fei Sun, Hua Jiang, Xing-jiang Long, Guo-hua Chen, Xin-yu Li, Chang-gang Li, Li-bin Huang, Ya-yun Ling, Dan-na Lin, Chuan Wen, Wen-yong Kuang, Xiao-qin Feng, Zhong-lv Ye, Bei-yan Wu, Xiang-lin He, Qiao-ru Li, Li-na Wang, Xian-ling Kong, Lu-hong Xu, Chi-kong Li, Jian-pei Fang

**Affiliations:** ^1^ Children’s Medical Center, Sun Yat-sen Memorial Hospital, Sun Yat-sen University, Guangzhou, China; ^2^ Guangdong Provincial Key Laboratory of Malignant Tumor Epigenetics and Gene Regulation, Sun Yat-Sen Memorial Hospital, Sun Yat-Sen University, Guangzhou, China; ^3^ Department of Paediatrics, Sun Yat-sen University First Affiliated Hospital, Guangzhou, China; ^4^ Department of Hematology and Oncology, Shenzhen Children’s Hospital, Shenzhen, China; ^5^ Department of Paediatrics, Guangxi Medical University First Affiliated Hospital, Nanning, China; ^6^ Department of Paediatrics, Southern Medical University Zhujiang Hospital, Guangzhou, China; ^7^ Department of Hematology, Hunan Children’s Hospital, Changsha, China; ^8^ Department of Paediatrics, Second Xiangya Hospital of Central South University, Changsha, China; ^9^ Department of Paediatrics, Southern Medical University Nanfang Hospital, Guangzhou, China; ^10^ Department of Paediatrics, Huizhou Central People’s Hospital, Huizhou, China; ^11^ Department of Paediatrics, First Affiliated Hospital of Nanchang University, Nanchang, China; ^12^ Department of Paediatrics, Third Affiliated Hospital of Sun Yat-sen University, Guangzhou, China; ^13^ Department of Paediatrics, Sun Yat-sen University Cancer Center, Guangzhou, China; ^14^ Department of Hematology, Guangzhou Women and Children’s Medical Center, Guangzhou, China; ^15^ Department of Paediatrics, Liuzhou People’s Hospital, Liuzhou, China; ^16^ Department of Paediatrics, Huizhou First People’s Hospital, HuiZhou, China; ^17^ Department of Paediatrics, Affiliated Hospital of Guangdong Medical University, Zhanjiang, China; ^18^ Department of Paediatrics, The First Affiliated Hospital of Shantou University Medical College, Shantou, China; ^19^ Department of Paediatrics, Hunan Provincial People’s Hospital, Changsha, China; ^20^ Department of Paediatrics, Zhongshan People’s Hospital, Zhongshan, China; ^21^ Department of Paediatrics, Guangzhou First People’s Hospital, Guangzhou, China; ^22^ Department of Paediatrics, Boai Hospital of Zhongshan, Zhongshan, China; ^23^ Department of Paediatrics, Hong Kong Children Hospital and Prince of Wales Hospital, The Chinese University of Hong Kong, Hong Kong, Hong Kong SAR, China

**Keywords:** ETV6/RUNX1, acute B lymphoblastic leukemia, outcome, prognosis, multicenter cohort study

## Abstract

**Purpose:**

To analyzed the outcome of ETV6/RUNX1-positive pediatric acute B lymphoblastic leukemia (B-ALL) with the aim of identifying prognostic value.

**Method:**

A total of 2,530 pediatric patients who were diagnosed with B-ALL were classified into two groups based on the ETV6/RUNX1 status by using a retrospective cohort study method from February 28, 2008, to June 30, 2020, at 22 participating ALL centers.

**Results:**

In total, 461 (18.2%) cases were ETV6/RUNX1-positive. The proportion of patients with risk factors (age <1 year or ≥10 years, WB≥50×10^9^/L) in ETV6/RUNX1-positive group was significantly lower than that in negative group (*P*<0.001), while the proportion of patients with good early response (good response to prednisone, D15 MRD < 0.1%, and D33 MRD < 0.01%) in ETV6/RUNX1-positive group was higher than that in the negative group (*P*<0.001, 0.788 and 0.004, respectively). Multivariate analysis of 2,530 patients found that age <1 or ≥10 years, SCCLG-ALL-2016 protocol, and MLL were independent predictor of outcome but not ETV6/RUNX1. The EFS and OS of the ETV6/RUNX1-positive group were significantly higher than those of the negative group (3-year EFS: 90.11 ± 4.21% *vs* 82 ± 2.36%, *P*<0.0001, 3-year OS: 91.99 ± 3.92% *vs* 88.79 ± 1.87%, *P*=0.017). Subgroup analysis showed that chemotherapy protocol, age, prednisone response, and D15 MRD were important factors affecting the prognosis of ETV6/RUNX1-positive children.

**Conclusions:**

ETV6/RUNX1-positive pediatric ALL showed an excellent outcome but lack of independent prognostic significance in South China. However, for older patients who have the ETV6/RUNX1 fusion and slow response to therapy, to opt for more intensive treatment.

## Introduction

Acute lymphoblastic leukemia (ALL) is the most common hematologic malignancy in children, accounting for 80% of childhood leukemia (1). At present, the results of long-term follow-up of pediatric ALL by several large-scale research centers show that the 5-year event-free survival (EFS) rate of childhood ALL is >80%, and the 5-year overall survival (OS) rate is higher than 90% (2–5).

The ETV6/RUNX1 (also known as TEL/AML1) gene fusion, created by the t (12;21) (p12;q22), is the most common translocation in childhood acute lymphoblastic leukemia (ALL), occurring in approximately 20–25% of B-ALL (6). Some reports demonstrated among patients with ALL representing ETV6/RUNX1-positive, there was an associated 5-year EFS rate of 80–97%, significantly higher than other subtypes, which revealed excellent outcome (7–10). Despite this, results from another study showed that a high incidence (20 to 24%) of the ETV6/RUNX1 fusion in relapsed patients with ALL, thereby casting doubt as to the prognostic significance of this genetic alteration (11).

Few multicenter cohort studies of pediatric patients with ETV6/RUNX1-positive ALL from China have been conducted. Whether the outcome of childhood ALL subtype is similar to that from other countries, and the important factors that influence outcome are largely unknown. In this study, we intend to use a multicenter, large-sample, retrospective cohort to analyze the outcome of ETV6/RUNX1-positive pediatric B-ALL over 10 years in South China with the aim of identifying significant prognostic variables.

## Patients and Methods

### Study Participants

A total of 2,530 pediatric patients (0–18 years old) who were diagnosed with B-ALL were recruited for this study from February 28, 2008, to June 30, 2020. Patients were then classified into two groups based on the ETV6/RUNX1 status by using a retrospective cohort study method. All patients were treated at one of 22 pediatric ALL collaborative centers as follows: Sun Yat-sen Memorial Hospital (n=538), Guangzhou Women and Children’s Medical Center (n=67), Sun Yat-sen University First Affiliated Hospital (n=290), Southern Medical University Nanfang Hospital (n=143), Shenzhen Children’s Hospital (n=436), Huizhou Central People’s Hospital (n=38), Third Affiliated Hospital of Sun Yat-sen University (n=68), Sun Yat-sen University Cancer Center (n=30), Guangdong Provincial People’s Hospital (n=1). Zhujiang Hospital (n=154), The First Affiliated Hospital of Shantou University Medical College (n=27), Huizhou First People’s Hospital (n=9), Boai Hospital of Zhongshan (n=5), Zhongshan People’s Hospital (n=22), Second Xiangya Hospital (n=127), First Affiliated Hospital of Nanchang University (n=42), Guangxi Medical University First Affiliated Hospital (n=270), Liuzhou People’s Hospital (n=43), Affiliated Hospital of Guangdong Medical University (n=52), Guangzhou First People’s Hospital (n=22), Hunan Children’s Hospital (n=122), Hunan Provincial People’s Hospital (n=24).

All inclusion and exclusion criteria are listed as follows: inclusion criteria (1): age ≤18 years (2); clinical presentation consistent with ALL and diagnosis of B-ALL based on morphological review of bone marrow smears, immunophenotyping, cytogenetics, and molecular genetics according to the WHO 2008 criteria (3); first-episode children. Exclusion criteria (1): T-lineage, mature B, and acute mixed leukemia; (2) secondary to immunodeficiency disease; (3) as a second malignancy; (4) Down’s syndrome; (5) glucocorticoid use for more than 1 week in the month before enrollment; (6) patients missing ETV6/RUNX1 data. The study was conducted in accordance with the principles set down in the Declaration of Helsinki and was approved by the Ethics Committee of Sun Yat-sen Memorial Hospital, and by ethics committee of other cooperation centers. All patients, or the patients’ parents/guardians, provided written informed consent. The trial is registered with the Chinese Clinical Trial Registry (Chi-CTR; https://www.chictr.org.cn/; number ChiCTR2000030357).

### Chemotherapy Protocol

Children diagnosed between February 2008 to September 2016 were treated according to the Guangdong Children’s Leukemia Group-ALL-2008 (GD-ALL-2008) protocol (n=981); and children diagnosed between October 2016 to June 2020 were treated according to the South China Children’s Leukemia Group-ALL-2016 (SCCLG-ALL-2016) protocol (n=1549).

The chemotherapeutic drug classes and the composition of the chemotherapy protocol at each time period were essentially the same for both regimens, i.e., diagnosis and assessment of sensitivity after 7 days of pretreatment with prednisone upon enrollment, continued initiation of VDLD (vincristine+dexamethasone+ L-asparaginase+daunorubicin) induced remission therapy, early intensive CAM (cyclophosphamide+cytarabine+6-mercaptopurine), mM (high-dose methotrexate + 6-mercaptopurine, or HR-1, HR-2, HR-3 all in two rounds), delayed intensive VDLD, CAM regimen (with 8 weeks of maintenance chemotherapy in between) in one or two rounds, and finally maintenance chemotherapy and regular intrathecal injections, with specific drug doses and risk assessment indicators in Ref (11, 12).

### Treatment Response

Early response to treatment was measured as the absolute number of peripheral lymphoblasts at Day-8 of induction therapy. Patients were classified as prednisone good responders (PGR) when the absolute peripheral lymphoblast count by induction Day 8 was less than 1,000/μl and as prednisone poor responders (PPR) when the count was 1,000/μl or higher. Morphological evaluation of bone marrow smear was performed on Day-15 and Day-33; and patients were classified according to their blast cells amount, with M1 (blast cells <5%), M2 (5 to <25%), or M3 (>25%).

Rapid early responders (RERs) had an M1 marrow by induction Day-15 and <0.1% minimal residual disease (MRD) in the Day-33 marrow by flow cytometry. Slow early responders (SERs) had an M1 marrow on induction Day-33 but with either an M2 or M3 marrow on induction Day-15 or MRD ≥ 0.1% on Day-33 marrow (13).

Complete remission (CR) was defined as less than 5% lymphoblasts in active hematopoietic BM in the absence of clinical evidence of disease at the end of induction. Relapse was defined as the presence of lymphoblasts (>25%) in the BM or on histological documentation of blasts in extramedullary sites after achievement of CR.

### Minimal Residual Disease Evaluation

Flow cytometry (FCM)-MRD was analyzed according to previous literature from French multicenter study groups for pediatric and adult ALL (14, 15). MRD was analyzed at the central protocol laboratory—the hematology labs of Kingmed Diagnostics Corperation by Kaluza software or Cellquest software. Reagents were provided from BD Biosciences (Becton, Dickinson, China) and Beckman Coulter Commercial Enterprise (China) Co., Ltd. MRD Day-15 positive was defined as MRD ≥ 0.1%, while MRD Day-33 positive was defined as MRD ≥ 0.01%.

### Follow-Up

All cases were followed up by outpatient review or telephone, with follow-up dates up to June 30, 2018, for children receiving the GD-ALL-2008 protocol and up to July 31, 2020, for children receiving the SCCLG-ALL-2016 protocol, with study endpoints set as death, lost to follow-up, or follow-up cutoff, and up to follow-up time for those lost to follow-up. Among the whole cohort, the median follow-up time was 2.6 years. The event-free survival (EFS) and overall survival (OS) of the study cohort were analyzed. EFS was defined as the time from diagnosis of ALL to the last follow-up in CR or the first event that included relapse, primary refractory disease, death, or secondary malignancy. OS was defined as the time from diagnosis of ALL to last follow-up or death from any cause. Treatment-Related Mortality (TRM) is death that occurs during chemotherapy without recurrence or secondary malignancy.

### Statistical Analyses

Baseline characteristics were grouped by ETV6/RUNX1 and presented as mean ± SD for continuous variables and as frequency (%) for categorical variables. Comparisons between groups were made using the chi-square test for categorical variables and analysis of variance or the Kruskal-Wallis test for continuous variables. Cox proportional hazards models were used to test the associations between EFS or OS and baseline covariates, with results presented as HRs with 95% CIs. Similarly, the HRs and 95% CIs of EFS or OS in each ETV6/RUNX1 subgroup were estimated, and their interactions were tested. Survival curves were generated using the Kaplan-Meier method and compared using the log-rank test. Adjusted HRs with 95% CIs were estimated to evaluate the association of variables and ETV6/RUNX1 and EFS or OS. A competing risk analysis was done to compare the association with different ETV6/RUNX1 status among relapse and death, where each cause was simultaneously modeled as a different event. We used a Gray test to compare the parameter estimates between causes of event. All statistical analyses were performed using the IBM SPSS Statistics version 22.0, and EmpowerStats (http://www.empowerstats.cn/). A 2-tailed p < 0.05 was considered to be statistically significant in all analyses.

## Results

### Baseline Characteristics of Pediatric ALL Patients

Eventually, 2,530 eligible participants were assigned to two groups based on the ETV6/RUNX1 status. The baseline characteristics of the eligible patients were presented in **Table 1**. Of them, 1,472 (58.2%) are male and 1,058 (41.8%) are female with a median age of 4.3 years old. In total, 981 patients accepted GD-ALL-2008 protocol, and 1,549 patients received SCCLG-ALL-2016 protocol. Among childhood ALL, the median of initial WBC was 9.3×10^9^/L, the median Hb was 76 g/L, and the median PLT was 53.0×10^9^/L. The entire cohort of children was tested for four fusion genes: ETV6/RUNX1, E2A/PBX1, MLL gene rearrangement (MLL-r), and BCR/ABL1. A total of 828 (32.7%) fusion genes were positive. Of these, 461 (18.2%) were positive for ETV6/RUNX1, 139 (5.5%) for E2A/PBX1, 91 (3.6%) for MLL-r, and 137 (5.4%) for BCR/ABL1.

**Table 1:** Baseline characteristics of study participants by ETV6/RUNX1 status classification.

Characteristics	Total	ETV6/RUNX1 status		*P* value
		Negative (n = 2,069)	Positive (n = 461)	
Gender, n (%)				0.852
Male	1,472 (58.2%)	1,202 (58.1%)	270 (58.6%)	
Female	1,058 (41.8%)	867 (41.9%)	191 (41.4%)	
Age (y), median (range)	4.3 (0.1–17.4)	4.4 (0.1–17.4)	4.2(0.8–14.2)	0.365
Age group (y)				<0.001
≥1, <10	2,176 (86.0%)	1,740 (84.1%)	436 (94.6%)	
≥10 or <1	354 (14.0%)	329 (15.9%)	25 (5.4%)	
Chemotherapy protocol, n (%)				0.279
SCCLG-ALL-2016 Protocol	1,549 (61.2%)	1,277 (61.7%)	272 (59.0%)	
GD-ALL-2008 Protocol	981 (38.8%)	792 (38.3%)	189 (41.0%)	
Initial WBC (×10^9^/L), median (range)	9.3 (0.1–1,095.0)	9.6 (0.2–1,095.0)	8.6 (0.1–268.5)	0.099
WBC group, n (%)				<0.001
<10×10^9^/L	1,306 (51.7%)	1,047 (50.7%)	259 (56.2%)	
≥10×10^9^/L, <50×10^9^/L	784 (31.0%)	633 (30.7%)	151 (32.8%)	
≥50×10^9^/L	436 (17.3%)	385 (18.6%)	51 (11.1%)	
Initial Hb (g/L), median (range)	76 (16–182.0)	77.0 (17–182.0)	73.5 (16.0–145.0)	<0.001
Hb group				<0.001
<60 g/L	530 (21.0%)	417 (20.2%)	113 (24.6%)	
≥60 g/L, <90 g/L	1,281 (50.8%)	1,028 (49.9%)	253 (55.0%)	
≥90 g/L, <110 g/L	507 (20.1%)	427 (20.7%)	80 (17.4%)	
≥110 g/L	204 (8.1%)	190 (9.2%)	14 (3.0%)	
Initial PLT (×10^9^/L), median (range)	53.0 (0.0–784.0)	90.0 (101.5) 53.0 (0.0–784.0)	75.0 (74.8) 52.0 (2.0–539.0)	0.190
PLT group				0.007
<100×10^9^/L	1,809 (71.7%)	1,464 (70.9%)	345 (75.0%)	
≥100, <300×10^9^/L	597 (23.7%)	491 (23.8%)	106 (23.0%)	
≥300×10^9^/L	118 (4.7%)	109 (5.3%)	9 (2.0%)	
Risk group, n (%)				<0.001
SR	674 (26.6%)	508 (24.6%)	166 (36.0%)	
IR	1,302 (51.5%)	1,052 (50.8%)	250 (54.2%)	
HR	554 (21.9%)	509 (24.6%)	45 (9.8%)	
Immunophenotype, n (%)				0.09
Pro-B	131 (5.2%)	112 (5.4%)	19 (4.1%)	
Common-B	1,722 (68.1%)	1,392 (67.3%)	330 (71.6%)	
Pre-B	161 (6.4%)	127 (6.1%)	34 (7.4%)	
Immature-B	516 (20.4%)	438 (21.2%)	78 (16.9%)	
CNSL, n (%)				0.193
Yes	79 (3.1%)	69 (3.3%)	10 (2.2%)	
No	2,451 (96.9%)	2,000 (96.7%)	451 (97.8%)	
BCR/ABL1 Status, n (%)				<0.001
Negative	2,390 (94.6%)	1,936 (93.6%)	454 (98.9%)	
Positive	137 (5.4%)	132 (6.4%)	5 (1.1%)	
MLL-r Status, n (%)				0.018
Negative	2,436 (96.4%)	1,985 (96.0%)	451 (98.3%)	
Positive	91 (3.6%)	83 (4.0%)	8 (1.7%)	
E2A/PBX1 Status, n (%)				<0.001
Negative	2,391 (94.5%)	1,935 (93.5%)	456 (98.9%)	
Positive	139 (5.5%)	134 (6.5%)	5 (1.1%)	
Prednisone Response, n (%)				<0.001
PGR	2,314 (92.1%)	1,871 (91.0%)	443 (96.9%)	
PPR	198 (7.9%)	184 (9.0%)	14 (3.1%)	
D15 BM, n (%)				0.788
M1	1,880 (78.1%)	1,540 (78.2%)	340 (77.6%)	
M2/M3	527 (21.9%)	429 (21.8%)	98 (22.4%)	
D33 BM, n (%)				0.004
M1	2,394 (98.1%)	1,948 (97.7%)	446 (99.8%)	
M2/M3	47 (1.9%)	46 (2.3%)	1 (0.2%)	
D15 MRD, n (%)				0.165
<0.1%	712 (40.8%)	573 (40.0%)	139 (44.3%)	
≥0.1%	1,034 (59.2%)	859 (60.0%)	175 (55.7%)	
D33 MRD, n (%)				0.01
<0.01%	1,441 (83.8%)	1,167 (82.7%)	274 (88.7%)	
≥0.01%	279 (16.2%)	244 (17.3%)	35 (11.3%)	

WBC, white blood cell counts; Hb, hemoglobin; PLT, platelet; CNSL, central nervous system leukemia; MLL-r, MLL rearrangement; BM, bone marrow; MRD, minimal residual disease.

### Comparison Between ETV6/RUNX1-Positive and ETV6/RUNX1-Negative ALL

ETV6/RUNX1-positive were present in 461 (18.2%) cases. Comparison between ETV6/RUNX1-positive and ETV6/RUNX1-negative ALL in terms of gender, age, risk group, routine blood test, early treatment response, and relapse are shown in **Table 1**. By comparing the two groups, no association was found in the sex ratio (*P*= 0.852). ETV6/RUNX1-positive patients had a median age of 4.2 years and a median presenting leukocyte count of 8.6×10^9^/L, whereas those with ETV6/RUNX1-negative had a median age of 4.4 years and a median presenting leukocyte count of 9.6×10^9^/L. The proportion of patients with risk factors (age <1 year or ≥10 years, WBC ≥50×10^9^/L) at initial diagnosis was significantly lower in the ETV6/RUNX1-positive group than in the negative group (5.4 *vs.* 15.9%, *P*< 0.001; 11.1 *vs.* 18.6%, *P*<0.001, respectively). The immunophenotype in the ETV6/RUNX1-positive group mostly occurred in Common-B, but not significantly different to the negative group (71.6% *vs* 67.3%, *P*= 0.137). The majority of ETV6/RUNX1-positive patients had combined moderate anemia and thrombocytopenia at the onset of disease, with a significantly higher proportion than the negative group (55 *vs.* 49.9%, *P*< 0.001; 75.0 *vs.* 70.9%, *P*= 0.007, respectively). ETV6/RUNX1-positive were detected in 9.8% of patients in the high-risk group, while negative cases were detected in 24.6% (*P*< 0.001).

### Early Response to Therapy

Prednisone response was available for 2,512 of the patients with known ETV6/RUNX1 status. Among them, 2,314 (92.1%) patients showed PGR, while 198 (7.9%) cases had PPR. Further comparing the prednisone response in the two groups (**Table 1**), the proportion of PGR in the ETV6/RUNX1-positive group was significantly higher than that in the negative group (96.9 *vs.* 91.0%, *P*<0.001). A total of 1,880 (78.1%) cases had an M1 marrow on induction Day-15, of which the ETV6/RUNX1- positive group was slightly lower than the negative group (77.6 *vs* 78.2%, *P*=0.788). As expected, 47 (1.9%) cases had either an M2 or M3, of which the ETV6/RUNX1-positive group was significantly lower than the negative group (0.2% vs. 2.3%, *P*=0.004). In total, 1,746 children in this study had their MRD measured in the Day-15 marrow by flow cytometry. Of the 1,746 patients, 712 (40.8%) experienced rapid early responses, and 1,034 (59.2%) were slow early responders. In addition, D-15 MRD positive in the ETV6/RUNX1-positive group were slightly lower than in the negative group (55.7 *vs* 60.0%, *P*=0.165). Finally, 1,720 children can be evaluated for D33-MRD, and 35 were positive for D33-MRD in the ETV6/RUNX1-positive group, while 244 cases were MRD positive in the negative group (11.3 *vs* 17.3%, *P*=0.01).

### Prognostic Significance of the Overall Cohort Among Pediatric ALL

Factors associated with a significantly elevated EFS and OS in pediatric patients with B-ALL from univariate analysis were age, gender, chemotherapy protocol, WBC, PLT, risk group, immunophenotype, BCR/ABL1 fusion gene, MLL-r fusion gene and ETV6/RUNX1, prednisone response, D15 bone marrow status, D33- BM, and D15-MRD (**Table 2**). Risk factors selected for univariate analysis that had a statistically significant impact on the children were included in the multivariate analysis. We identified that SCCLG-ALL-2016 protocol, MLL-r positive, age<1 years or ≥10 years, and WBC >50×10^9^/L were independent factors for EFS or OS (**Table 3**).

**Table 2:** Univariate and analysis for EFS and OS among pediatric patients with B-ALL.

Variables	EFS		OS	
	*HR* (95%*CI*)	*P value*	*HR* (95%*CI*)	*P value*
Gender				
Male	Ref.		Ref.	
Female	0.7 (0.6, 0.9)	0.013	0.9 (0.7, 1.2)	0.446
Age group				
≥1y, <10y	Ref.		Ref.	
≥10y or <1y	2.2 (1.7, 2.9)	<0.001	2.3 (1.6, 3.2)	<0.001
Chemotherapy protocol				
SCCLG-ALL-2016 Protocol	Ref.		Ref.	
GD-ALL-2008 Protocol	1.9 (1.5, 2.4)	<0.001	2.1 (1.5, 2.8)	<0.001
WBC group				
<10×10^9^/L	Ref.		Ref.	
≥10×10^9^/L, <50×10^9^/L	1.5 (1.1, 1.9)	0.006	1.5 (1.1, 2.1)	0.015
≥50×10^9^/L	2.3 (1.7, 3.1)	<0.001	2.4 (1.7, 3.4)	<0.001
Hb group				
<60 g/L	Ref.		Ref.	
≥60 g/L, <90 g/L	1.2 (0.9, 1.6)	0.263	1.2 (0.8, 1.8)	0.293
≥90 g/L, <110 g/L	1.2 (0.8, 1.7)	0.344	1.0 (0.6, 1.5)	0.839
≥110 g/L	1.1 (0.6, 1.8)	0.815	1.0 (0.5, 1.8)	0.921
PLT group				
<100×10^9^/L	Ref.		Ref.	
≥100, <300×10^9^/L	0.6 (0.4, 0.8)	<0.001	0.6 (0.4, 0.9)	0.007
≥300×10^9^/L	0.9 (0.5, 1.6)	0.690	0.5 (0.2, 1.2)	0.134
Risk group				
SR	Ref.		Ref.	
IR	1.4 (1.0, 2.0)	0.029	1.9 (1.2, 3.0)	0.004
HR	3.4 (2.4, 4.7)	<0.001	4.8 (3.1, 7.6)	<0.001
Immunophenotype				
Pro-B	Ref.		Ref.	
Common-B	0.6 (0.4, 0.9)	0.028	0.6 (0.3, 1.0)	0.033
Pre-B	0.6 (0.3, 1.1)	0.118	0.5 (0.2, 1.1)	0.099
Immature-B	0.8 (0.5, 1.3)	0.403	0.6 (0.4, 1.1)	0.126
CNSL				
Yes	Ref.		Ref.	
No	1.0 (0.5, 2.0)	0.986	1.2 (0.5, 2.9)	0.705
BCR/ABL1 Status				
Negative	Ref.		Ref.	
Positive	1.7 (1.1, 2.7)	0.011	1.8 (1.1, 3.1)	0.020
MLL-r Status				
Negative	Ref.		Ref.	
Positive	2.8 (1.9, 4.2)	<0.001	2.9 (1.8, 4.8)	<0.001
E2A/PBX1 Status				
Negative	Ref.		Ref.	
Positive	0.9 (0.5, 1.6)	0.722	0.8 (0.4, 1.6)	0.512
ETV6/RUNX1 Status				
Negative	Ref.		Ref.	
Positive	0.5 (0.3, 0.7)	<0.001	0.6 (0.4, 0.9)	0.019
Prednisone Response				
PGR	Ref.		Ref.	
PPR	2.3 (1.6, 3.2)	<0.001	3.2 (2.2, 4.6)	<0.001
D15 BM				
M1	Ref.		Ref.	
M2/M3	2.4 (1.9, 3.1)	<0.001	2.7 (2.0, 3.6)	<0.001
D33 BM				
M1	Ref.		Ref.	
M2/M3	2.6 (1.3, 5.0)	0.005	3.7 (1.8, 7.6)	<0.001
D15 MRD				
<0.1%	Ref.		Ref.	
≥0.1%	1.7 (1.2, 2.4)	0.005	2.1 (1.3, 3.5)	0.003
D33 MRD				
<0.01%	Ref.		Ref.	
≥0.01%	1.1 (0.7, 1.8)	0.587	1.2 (0.7, 2.1)	0.522

WBC, white blood cell counts; Hb, hemoglobin; PLT, platelet; CNSL, central nervous system leukemia; MLL-r, MLL rearrangement; BM, bone marrow; MRD, minimal residual disease.

**Table 3:** Multivariate analysis for EFS and OS among pediatric patients with B-ALL.

Outcome	Variable	*HR* (95% *CI*)	*P* value
EFS	SCCLG-ALL-2016 Protocol	0.5 (0.3, 0.8)	0.001
	Age ≥10y or <1y	2.0 (1.3, 3.2)	0.002
	WBC ≥50×10^9^/L	2.0 (1.1, 3.4)	0.016
	HR	2.2 (1.0, 4.9)	0.051
	MLL-r(+)	1.6 (0.7, 3.7)	0.258
	ETV6/RUNX1(+)	0.7 (0.4, 1.2)	0.194
	PPR	0.8 (0.4, 1.5)	0.486
	D15 BM	1.4 (0.9, 2.3)	0.181
	D33 BM	0.7 (0.2, 2.4)	0.609
	D15 MRD	1.1 (0.7, 1.8)	0.694
	D33 MRD	0.9 (0.5, 1.5)	0.636
OS	SCCLG-ALL-2016 Protocol	0.6 (0.3, 1.1)	0.124
	Age ≥10y or <1y	2.0 (1.1, 3.7)	0.026
	WBC ≥50×10^9^/L	1.6 (0.7, 3.4)	0.235
	HR	2.8 (0.8, 9.5)	0.098
	MLL-r(+)	3.0 (1.0, 8.7)	0.042
	ETV6/RUNX1(+)	0.9 (0.4, 1.9)	0.733
	PPR	1.8 (0.8, 4.2)	0.147
	D15 BM	1.5 (0.8, 3.0)	0.197
	D33 BM	1.5 (0.4, 5.3)	0.553
	D15 MRD	1.1 (0.5, 2.1)	0.858
	D33 MRD	0.9 (0.5, 1.9)	0.843

WBC, white blood cell counts; HR, high risk; PPR, prednisone poor responders; MLL-r, MLL rearrangement; BM, bone marrow; MRD, minimal residual disease.

As shown in **Table 3**, SCCLG-ALL-2016 protocol was significantly associated with better EFS (*HR*=0.5, 95% *CI*: 0.3–0.8, *P*<0.001), while WBC>50×10^9^/L revealed a significant decrease in EFS (*HR*=2.0, 95% *CI*: 1.1–3.4, *P*=0.016). Those patients with MLL-r positive were also significantly associated with worse EFS (*HR*=3.0, 95% *CI*: 1.0–8.7, *P*=0.042). Age<1 years or ≥10 years was significantly associated with EFS (*HR*=2.0, 95% *CI*: 1.3–3.2, *P*=0.002) and OS (*HR*=2.0, 95% *CI*: 1.1–3.7, *P*=0.026). There was a trend towards an independent prognostic effect in the high-risk group (EFS: *HR*=2.2, 95% *CI*: 1.0–4.9, *P*=0.051). In contrast, ETV6/RUNX1-positive was not an independent prognostic influence (EFS: *HR*=0.7, 95% *CI*: 0.4–1.2, *P*=0.194; OS: *HR*=0.9, 95% *CI*: 0.4–1.9, *P*=0.733).

**Figure 1:**
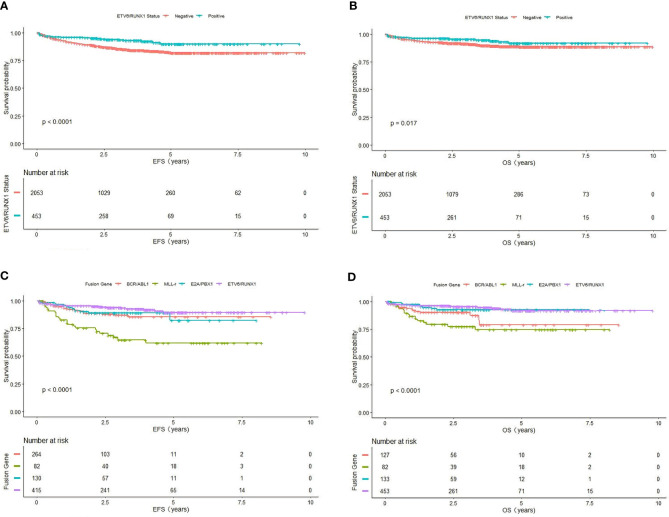
Survival cures of fusion gene. **(A)** The cumulative event-free survival of ETV6/RUNX1-positive and ETV6/RUNX1-negative patients. **(B)** The cumulative overall survival of ETV6/RUNX1-positive and ETV6/RUNX1-negative patients. **(C)** The cumulative event-free survival of four fusion gene patients. **(D)** The cumulative overall survival of four fusion gene patients.

Further comparison of the K-M survival curves of 461 ETV6/RUNX1-positive and 2,069 ETV6/RUNX1-negative patients showed that the estimate EFS rate and OS rate were significantly higher in ETV6/RUNX1-positive than in negative patients (3-year EFS: 90.11 ± 4.21% *vs* 82 ± 2.36%, *P*<0.0001; 3-year OS: 91.99 ± 3.92% *vs* 88.79 ± 1.87%, *P*= 0.017) (**Figures 1A, B**). Among the 828 children with positive fusion genes, the estimate EFS for ETV6/RUNX1-positive, E2A/PBX1-positive, MLL-r-positive, and BCR/ABL1-positive children were 90.11 ± 4.21%, 81.87 ± 12.81%, 60.65 ± 11.68%, and 74.88 ± 10.85%, respectively (*P*<0.0001) (**Figure 1C**); estimate OS was 91.99 ± 3.92%, 90 ± 2%, 74.53 ± 10.28%, and 79.14 ± 11.92% (*P*<0.0001), respectively (**Figure 1D**).

**Figure 2:**
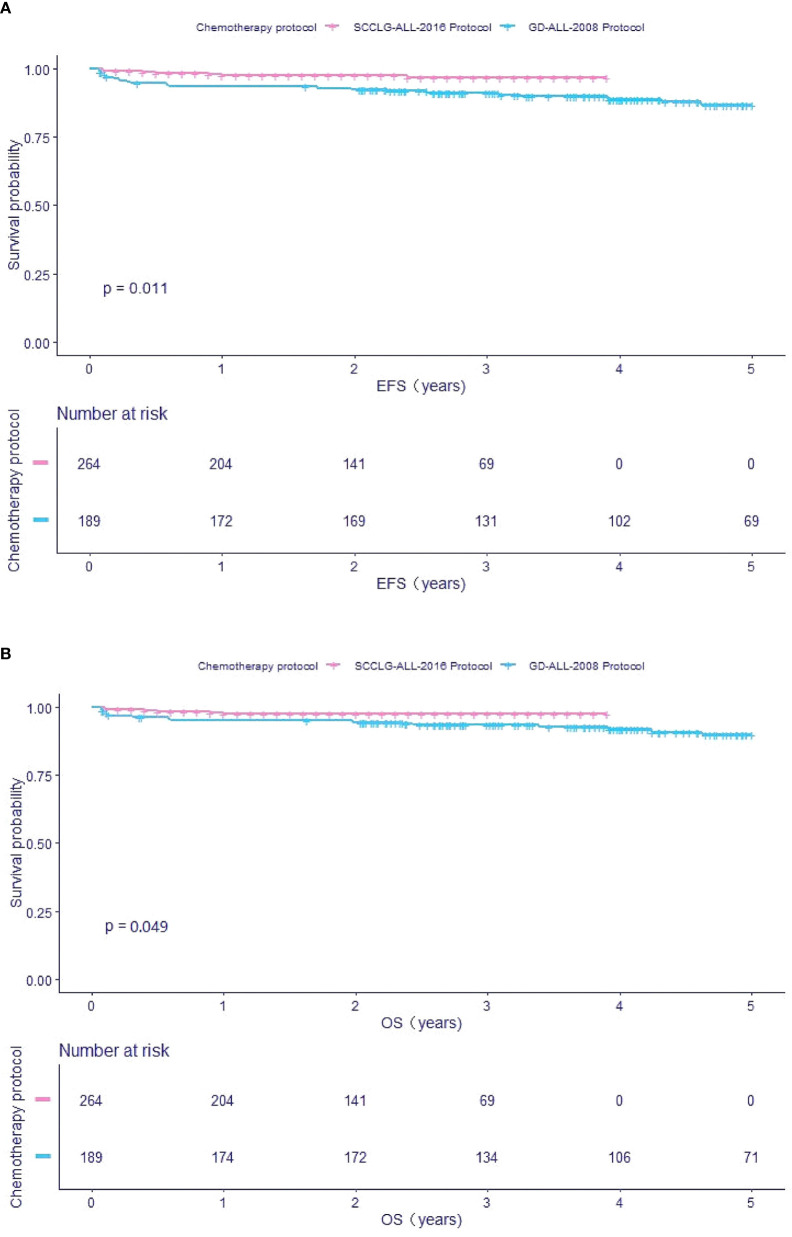
Survival cures of ETV6/RUNX1-positive pediatric B-ALL with different chemotherapy protocol. **(A)** The cumulative event-free survival of GD-ALL-2008 and SCCLG-ALL-2016 patients. **(B)** The cumulative overall survival of GD-ALL-2008 and SCCLG-ALL-2016 patients.

Comparing the survival rate of different chemotherapy protocols with ETV6/RUNX1-positive children, we found that the estimate EFS and OS in pediatric patients receiving the SCCLG-ALL-2016 protocol were significantly higher than in those receiving the GD-ALL-2008 protocol (EFS: 96.64 ± 2.2% *vs* 88.97 ± 4.62%, *P*=0.011; OS: 97.48 ± 1.99% *vs* 91.82 ± 4.07%, *P*=0.049, respectively) (**Figures 2A, B**).

### Relapse Analysis

**Figure 3:**
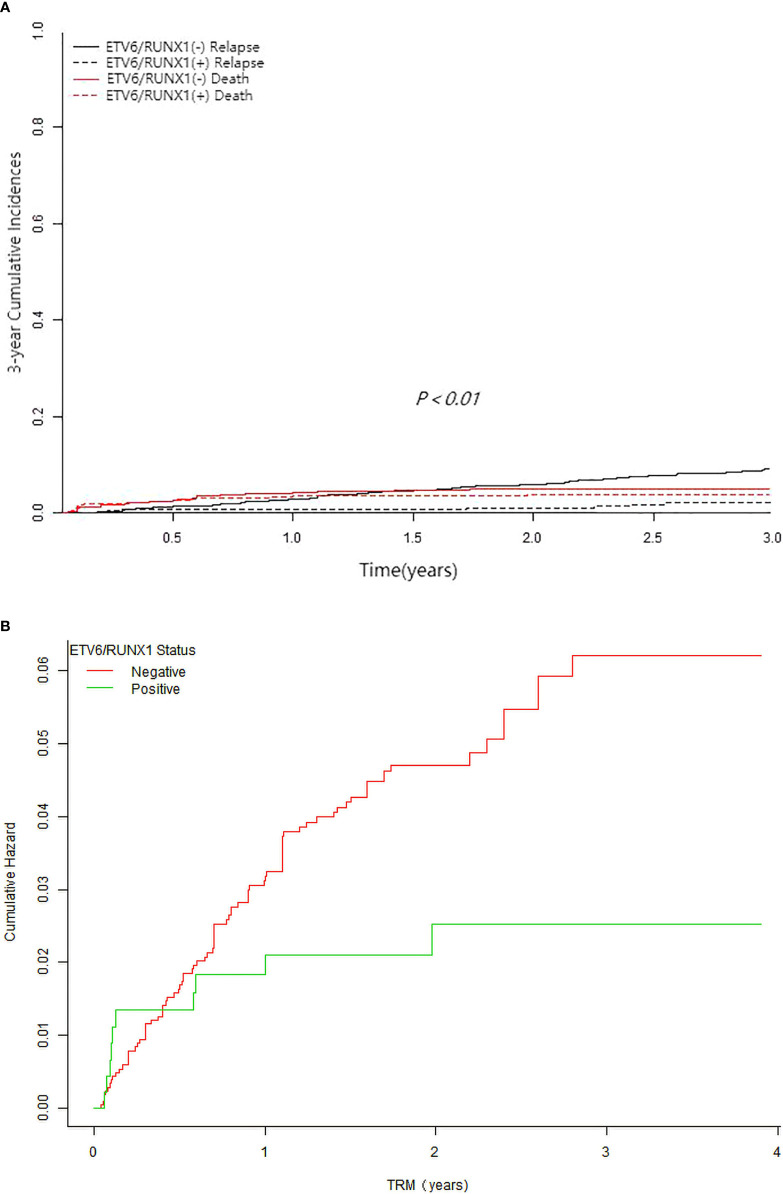
Survival cures of pediatric B-ALL with different ETV6/RUNX1 status. **(A)** The 3-year cumulative incidence of relapse and deaths of ETV6/RUNX1-positive and ETV6/RUNX1-negative for competing risks. **(B)** The cumulative incidence of treatment-related mortality of ETV6/RUNX1-positive and ETV6/RUNX1-negative.

Follow-up analysis indicated that 178 (7%) children relapsed, predominantly with BM relapse alone (n=130). Among them, ETV6/RUNX1-negative group had 161 relapse and ETV6/RUNX1-positive group had 17 relapse (3 early relapses and 14 mid-to late-stage relapses) (7.8 *vs* 3.7%, *P*< 0.001). In the ETV6/RUNX1-positive group, after taking into account the competitive risk of death, the 3-year estimate CIR rate was significantly lower (2.04 ± 0.88% *vs* 9.2 ± 1.32%, *P*<0.01) (**Figure 3A**), with a significantly lower estimate TRM (2.49 ± 1.55 *vs* 6.01 ± 1.42%, *P*=0.033) (**Figure 3B**) when compared to the ETV6/RUNX1-negative group. Furthermore, comparing the time to relapse between the two groups, the median time to relapse was 1.8 years in the ETV6/RUNX1-negative group and 2.8 years in the ETV6/RUNX1-positive group (*P*=0.048).

### Subgroup Analysis of Prognostic Significance for ETV6/RUNX1 Patients

**Figure 4:**
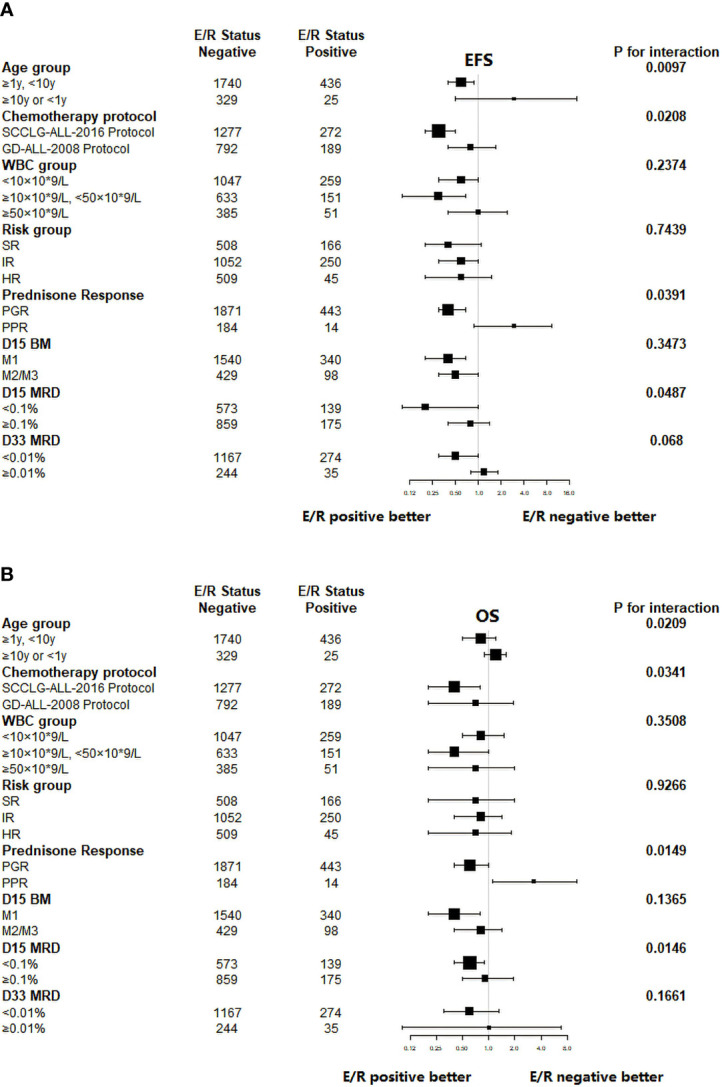
Subgroup analysis of the associations between ETV6/RUNX1 status and outcome. **(A)** Hazard Ratios for EFS in pediatric patients in the ETV6/RUNX1-positive and ETV6/RUNX1-negative group according the risk factors. **(B)** Hazard Ratios for OS in pediatric patients in the ETV6/RUNX1-positive and ETV6/RUNX1-negative group according the risk factors. E/R for ETV6/RUNX1.

Further stratified analysis of long-term survival was performed according to age, chemotherapy protocol, WBC, risk classification, and early treatment response depending on the expression of ETV6/RUNX1 fusion gene. Then, the correlation between each stratification factor and ETV6/RUNX1 and prognosis (EFS or OS) respectively was tested for interaction and adjusted for relevant confounders to obtain the modification effect of the presence of variables: chemotherapy protocol, age, prednisone response, and D-15 MRD (*P*<0.05) (**Tables 4**, **5**). The results showed that ETV6/RUNX1-positive cases on the SCCLG-ALL-2016 protocol, age ≥1 years and <10 years, and PGR and D-15 MRD negative had better EFS and OS than those on the GD-ALL-2008 protocol, age <1 or ≥10 years, and PPR and D-15 MRD positive, respectively (**Figures 4A, B**).

**Table 4:** Subgroup analysis of the associations between ETV6/RUNX1 status and EFS.

Subgroup	ETV6/RUNX1 status		*Crude HR* (95%CI)	Adjusted*HR* (95%CI)	*P value*	*P* for interaction
	Negative	Positive				
Age group						0.0097
≥1y, <10y	1,740	436	0.6 (0.4, 0.9)	0.6 (0.4, 0.9)	0.011	
≥10y or <1y	329	25	3.0 (0.5, 20.2)	3.0 (0.5, 20.2)	0.249	
Chemotherapy protocol						0.0208
SCCLG-ALL-2016 Protocol	1,277	272	0.3 (0.2, 0.7)	0.3 (0.2, 0.5)	<0.001	
GD-ALL-2008 Protocol	792	189	0.8 (0.5, 1.4)	0.8 (0.4, 1.7)	0.588	
WBC group						0.2374
<10×10^9^/L	1,047	259	0.5 (0.3, 0.9)	0.6 (0.3, 1.0)	0.045	
≥10×10^9^/L, <50×10^9/^L	633	151	0.3 (0.1, 0.7)	0.3 (0.1, 0.7)	0.003	
≥50×10^9^/L	385	51	0.8 (0.4, 1.8)	1.0 (0.4, 2.4)	0.969	
Risk group						0.7439
SR	508	166	0.4 (0.2, 1.0)	0.4 (0.2, 1.1)	0.079	
IR	1,052	250	0.6 (0.4, 1.0)	0.6 (0.3, 1.0)	0.053	
HR	509	45	0.5 (0.2, 1.3)	0.6 (0.2, 1.5)	0.283	
Prednisone Response						0.0391
PGR	1,871	443	0.4 (0.3, 0.7)	0.4 (0.3, 0.7)	<0.001	
PPR	184	14	1.7 (0.6, 4.7)	3.0 (0.9, 9.5)	0.062	
D15 BM						0.3473
M1	1,540	340	0.4 (0.2, 0.7)	0.4 (0.2, 0.7)	0.001	
M2/M3	429	98	0.5 (0.3, 0.9)	0.5 (0.3, 1.0)	0.038	
D33 BM						_§
M1	1,948	446	0.5 (0.3, 0.7)	0.5 (0.3, 0.7)	<0.001	
M2/M3	46	1	_§	_§	_§	
D15 MRD						0.0487
<0.1%	573	139	0.5 (0.3, 0.7)	0.2 (0.1, 1.0)	0.046	
≥0.1%	859	175	0.7 (0.4, 1.3)	0.8 (0.4, 1.4)	0.397	
D33 MRD						0.068
<0.01%	1,167	274	0.5 (0.3, 1.0)	0.5 (0.3, 1.0)	0.059	
≥0.01%	244	35	1.8 (1.2, 2.6)	1.2 (0.8, 1.8)	0.327	

§The model failed because of the small sample size.

WBC, white blood cell counts; SR, standard risk; IR, intermediate risk; HR, high risk; PPR, prednisone poor responders; PGR, prednisone good responders; BM, bone marrow; MRD, minimal residual disease.

The column “crude HR” of **Table 4** reports the results of the Cox models (for HR of ETV6/RUNX1 pos vs ETV6/RUNX1 neg) within subgroups defined by levels of the various prognostic factors. And the “adjusted HR” column indeed reports the results of the Cox model including the interaction term.

**Table 5:** Subgroup analysis of the associations between ETV6/RUNX1 status and OS.

Subgroup	ETV6/RUNX1 status		Crude *HR* (95%CI)	Adjusted*HR* (95%CI)	*P value*	*P* for interaction
	Negative	Positive				
Age group						0.0209
≥1y, <10y	1,740	436	0.7 (0.5, 1.2)	0.8 (0.5, 1.2)	0.239	
≥10y or <1y	329	25	1.8 (0.3, 10.8)	1.2 (0.9, 1.6)	0.185	
Chemotherapy protocol						0.0341
SCCLG-ALL-2016 Protocol	1,277	272	0.4 (0.2, 1.1)	0.4 (0.2, 0.8)	0.007	
GD-ALL-2008 Protocol	792	189	0.7 (0.4, 1.2)	0.7 (0.2, 1.9)	0.489	
WBC group						0.3508
<10×10^9^/L	1,047	259	0.9 (0.5, 1.6)	0.8 (0.5, 1.5)	0.570	
≥10×10^9^/L, <50×10^9/^L	633	151	0.4 (0.1, 0.9)	0.4 (0.2, 1.0)	0.042	
≥50×10^9^/L	385	51	0.6 (0.2, 1.8)	0.7 (0.2, 2.0)	0.481	
Risk group						0.9266
SR	508	166	0.7 (0.2, 1.9)	0.7 (0.2, 2.0)	0.494	
IR	1,052	250	0.8 (0.4, 1.5)	0.8 (0.4, 1.4)	0.412	
HR	509	45	0.6 (0.2, 1.7)	0.7 (0.2, 1.8)	0.434	
Prednisone Response						0.0149
PGR	1,871	443	0.6 (0.3, 0.9)	0.6 (0.4, 1.0)	0.033	
PPR	184	14	2.3 (0.8, 6.5)	3.3 (1.1, 10.4)	0.036	
D15 BM						0.1365
M1	1,540	340	0.4 (0.2, 0.8)	0.4 (0.2, 0.8)	0.012	
M2/M3	429	98	0.7 (0.4, 1.4)	0.8 (0.4, 1.4)	0.381	
D33 BM						_§
M1	1,948	446	0.6 (0.4, 1.0)	0.6 (0.4, 1.0)	0.066	
M2/M3	46	1	_§	_§	_§	
D15 MRD						0.0146
<0.1%	573	139	0.6 (0.4, 1.0)	0.6 (0.4, 0.9)	0.007	
≥0.1%	859	175	1.0 (0.5, 2.1)	0.9 (0.5, 1.9)	0.859	
D33 MRD						0.1661
<0.01%	1,167	274	0.7 (0.3, 1.4)	0.6 (0.3, 1.3)	0.229	
≥0.01%	244	35	1.6 (1.0, 2.5)	1.0 (0.1, 6.8)	0.961	

§The model failed because of the small sample size.

Adjusted: Adjusted for: Gender, Cell of Origin, CNS Disease, MLL Status, BCR/ABL1 Status, E2A/PBX1 Status, Hb group, PLT group.

WBC, white blood cell counts; SR, standard risk; IR, intermediate risk; HR, high risk; PPR, prednisone poor responders; PGR, prednisone good responders; BM, bone marrow; MRD, minimal residual disease.

The column “crude HR” of **Table 4** reports the results of the Cox models (for HR of ETV6/RUNX1 pos vs ETV6/RUNX1 neg) within subgroups defined by levels of the various prognostic factors. And the “adjusted HR” column indeed reports the results of the Cox model including the interaction term.

## Discussion

The proportion of children with ETV6/RUNX1-positive ALL in this study was 18.2% of ALL in the same period, which is similar to the results in Korea (16) (23.1%), Greece (17) (22.7%), Czech Republic (18) (22%), Turkey (19) (25.5%), and the United States (20) (12.8%), but higher than India (14) (6%), Mexico (15) (9.6%) and lower than Iran (21) (34.9) and Europe (22) (31.5%). The exact reasons for the differences in rates across countries are unclear, and we speculate that they may be due to ethnic differences.

Clinical characteristics of our cohort of ETV6/RUNX1-positive ALL children included age at childhood onset mostly distributed between 1 and 10 years (94.6%), with a median age of 4.2 years and no infantile leukemia; median initial WBC of 8.6 × 10^9^/L and <10 × 10^9^/L in 56.2%; mostly combined with moderate anemia and thrombocytopenia; immunophenotype of common B-ALL was predominant (71.6%); the risk classification was only 9.8% in the high-risk group, which is largely consistent with what has been reported in most previous studies (14, 15, 17–23). It has been clinically reported that ETV6/RUNX1-positive children were found to have a higher proportion with good early treatment response in addition to fewer risk factors at initial diagnosis (24, 25). In the present study, it was also observed that the prednisone response and bone marrow MRD on Day 33 of induction chemotherapy were better in ETV6/RUNX1-positive children with ALL than in ETV6/RUNX1-negative cases, indicating a good early treatment response and a high rate of bone marrow CR in ETV6/RUNX1-positive children. This could be related to the unique biological characteristics of the ETV6/RUNX1 gene: the ETV6/RUNX1 gene can overcome chemoresistance by transcriptionally repressing the expression of the multidrug resistance-1 gene; ETV6/RUNX1-positive leukemic cells have increased sensitivity to anti-leukemic drugs *in vitro* compared to other cytogenetic subtypes of leukemic cells (26–30).

In this present study, comparison revealed that the 10-year estimate EFS and OS of the 461 ETV6/RUNX1-positive children in this study were 90.11 ± 4.21 and 91.99 ± 3.92%, both significantly higher than 82 ± 2.36% and 88.79 ± 1.87% of the 2,069 ETV6/RUNX1-negative patients, indicating that the overall prognosis of ETV6/RUNX1-positive children was better, consistent with the above study report. Several large sample size studies have shown that the prognosis of ETV6/RUNX1-positive childhood ALL is better. Rubnitz et al. (23) analyzed the prognosis of 244 ETV6/RUNX1-positive and 682 ETV6/RUNX1-negative children with B-ALL and showed that the EFS rate was significantly higher in the former than in the latter (82 ± 2% *vs* 72 ± 2%, *P* < 0.001), and ETV6/RUNX1-positive was an independent prognostic factor. Bhojwani et al. (9) compared the prognosis of 168 ETV6/RUNX1-positive and 494 ETV6/RUNX1-negative B-ALL cases and showed that 5-year EFS and OS rates were significantly higher in the former than in the latter.

The results of the multivariate analysis in this study showed that age, WBC, MLL-r gene, and chemotherapy regimen were all independent prognostic factors affecting the prognosis of the B-ALL patients. Age, WBC, and MLL-r gene have been reported to be strongly associated with prognosis in children with B-ALL, all of which can be used as a basis for the risk classification of pediatric ALL (16, 31–33). The SCCLG-ALL-2016 protocol currently used by our collaborative group is also considered an independent influence factor on good prognosis, which could be explained by the fact that relative to the GD-ALL-2008 protocol, the SCCLG-ALL-2016 protocol uses monitoring of MRD as a risk adjustment in addition to a comprehensive risk stratification based on clinical and biogenetic characteristics and treatment response to guide treatment. As a result, the incidence of adverse events and mortality in children with ALL were significantly reduced due to the timely change of treatment regimens of different intensities.

To identify poor prognosticators among ETV6/RUNX1-positive children early, we found by subgroup analysis that the association between ETV6/RUNX1-positive children and prognosis was significantly influenced by chemotherapy protocol, age, PR, and MRD on day 15 of induction chemotherapy. Some findings (9, 10, 16, 23) have shown that the ETV6/RUNX1-positive ALL has favorable outcome and was one of the independent prognostic factors. However, a retrospective study of 105 children, 22 of whom were positive for the ETV6/RUNX1 gene treated with the Dutch Collaborative Childhood Leukemia Study Group-VIII protocol (DCLSG-VIII), found that this fusion gene is an independent prognostic factor that was lacking in children treated with DCLSG-VIII, and children with this subtype did not have a better prognosis than other subtypes (7). The COX regression model in this study showed that ETV6/RUNX1 was not an independent prognostic factor affecting children with B-ALL, in agreement with the findings of the Dutch Leukemia Collaborative Group.

Our K-M survival analysis also showed that ETV6/RUNX1-positive children receiving the SCCLG-ALL-2016 protocol had a 5-year estimate EFS and OS of 96.64 ± 2.2% and 97.48 ± 1.99%, respectively, significantly higher than those of GD-ALL-2008 protocol positive case at 88.97 ± 4.62% and 91.82 ± 4.07%, indicating that the SCCLG-ALL-2016 protocol is more suitable for ETV6/RUNX1-positive children and significantly improves the prognosis. However, it should be noted that the median follow-up of the SCCLG-ALL-2016 protocol in this study was less than 5 years, and it has been reported in the literature (8) that ETV6/RUNX1-positive pediatric patients are prone to late relapses, with 80% of relapsed cases presenting 6 years after diagnosis carrying the ETV6/RUNX1 fusion gene.

In addition, the prognosis of older (≥ 10 years) ETV6/RUNX1-positive patients were worse than younger ones, which was mainly related to the insensitivity of older children to chemotherapeutic drugs and their susceptibility to drug resistance. Usami et al. (34) found that interruption of menadione enzyme therapy and poor response to prednisone pretreatment were poor prognostic factors in ETV6/RUNX1-positive children. It has also been more widely reported in the literature (10, 35, 36) that MRD levels were the most important poor prognostic indicator affecting ETV6/RUNX1-positive cases. Pui et al. (37) showed that MRD at day 19 of induction had a significant impact on ETV6/RUNX1-positive B-ALL and had a guiding prognostic judgment, and a subsequent research confirmed that children with MRD ≥ 0.1% were at greater risk of treatment failure or relapse. Our study also observed a relatively poor prognosis for ETV6/RUNX1-positive children with PPR and D15-MRD ≥0.1%, suggesting a poor prognosis for those with a slow response to early treatment. Therefore, clinicians should intervene early in this group of ETV6/RUNX1-positive children by promptly adjusting the risk classification and recommending them to receive more intense chemotherapy. The difference in results between our study and other different study centers also suggested that for ETV6/RUNX1-positive children with B-ALL, the risk stratification criteria and choice of treatment regimen can significantly affect the prognosis of such children and is a key factor to further improve the prognosis with this subtype (38).

In the present study, the proportion of relapses and 10-year predicted CIR rates were significantly lower in ETV6/RUNX1-positive children than in the ETV6/RUNX1-negative group, with only 13 relapses (3 early relapses and 10 mid- to late-term relapses). And as far as the time point of recurrence in ETV6/RUNX1-negative children was concerned, the time of recurrence in ETV6/RUNX1-positive children was significantly later than that in ETV6/RUNX1-negative children. A study (39) found that relapses in ETV6/RUNX1-positive children were basically late relapses more than 3 years from the initial diagnosis, which is consistent with the results of the present study. For the reason of late relapse in ETV6/RUNX1-positive children, it has been suggested (40) that in some children, combination chemotherapy may fail to eliminate the clone of the pre-fetal leukemia ETV6/RUNX1 gene and that secondary transformation of this clone occurs after treatment leading to leukemic relapse. Of the 13 children with ETV6/RUNX1-positive relapses explored further, 6 are currently alive, while the remaining 7 eventually died from disease progression despite treatment with relapse regimens. We also found that the seven children who died had both PPR and D15 MRD ≥ 0.1%, which parallel with our previous findings that ETV6/RUNX1-positive children with slow response to early treatment have a poorer prognosis. However, as there were only 13 recurrent cases in the current study, this remains to be determined after further expansion of the sample size. In addition to treatment efficacy, the adverse events of treatment in ETV6/RUNX1-positive children should also be of concern. Our study showed that the 4-year predicted TRM in ETV6/RUNX1-positive children was significantly lower than that in ETV6/RUNX1-negative individuals, which may be due to the fact that ETV6/RUNX1-positive children have fewer initial risk factors and better early treatment response.

In summary, our results demonstrate that chemotherapy protocol, age, WBC, and mll-r status are all independent, significant predictors of outcome among childhood B-ALL but not ETV6/RUNX1 status. ETV6-RUNX1-positive B-ALL children have fewer risk factors at diagnosis, better early response, lower recurrence rate, and good prognosis than that of ETV6-RUNX1-negative B-ALL children. Positive children are more likely to have a mid- to late-stage recurrence, and those with recurrence have a poor prognosis and should be followed up over time. However, the prognosis of ETV6-RUNX1-positive B-ALL was significantly affected by age, prednisone response, D15 MRD, and chemotherapy protocol. Our study revealed the SCCLG-ALL-2016 regimen is a good choice for children with ETV6/RUNX1-positive ALL. As mentioned above, we suggest that patients who have ETV6/RUNX1-positive slow responses to therapy to opt for more intensive treatment.

## Data Availability Statement

The raw data supporting the conclusions of this article will be made available by the authors, without undue reservation.

## Ethics Statement

The studies involving human participants were reviewed and approved by the Ethics Committee of Sun Yat-sen Memorial Hospital. Written informed consent to participate in this study was provided by the participants’ legal guardian/next of kin.

## Author Contributions

K-yQ, H-gX, X-qL, H-rM, NL, L-hY, M-cZ, W-qW, and X-dW wrote the manuscript. R-yL, Q-wC, H-qC, X-fS, HJ, X-jL, G-hC, X-yL, C-gL, L-bH, Y-yL, and D-nL for support and analysis of data. CW, W-yK, X-qF, Z-lY, B-yW, X-lH, Q-rL, L-nW, X-lK, L-hX, and C-kL polished the language. J-pF reviewed the manuscript and supported the funding. All authors contributed to the article and approved the submitted version.

## Funding

This work was supported by the Guangzhou Science and Technology Program key projects (No. 201803010032) and Bethune Medical Scientific Research Fund Project (No. SCE111DS). The study was supported by grant from Guangdong Science and Technology Department (2020B1212060018).

## Conflict of Interest

The authors declare that the research was conducted in the absence of any commercial or financial relationships that could be construed as a potential conflict of interest.

## Publisher’s Note

All claims expressed in this article are solely those of the authors and do not necessarily represent those of their affiliated organizations, or those of the publisher, the editors and the reviewers. Any product that may be evaluated in this article, or claim that may be made by its manufacturer, is not guaranteed or endorsed by the publisher.
