# Supplementary material for: Prognostic Value and Outcome for ETV6/RUNX1-Positive Pediatric Acute Lymphoblastic Leukemia: A Report From the South China Children’s Leukemia Group
Source: Front Oncol. 2021 Dec 20;11:797194. doi: 10.3389/fonc.2021.797194 (PMC8722219; doi:10.3389/fonc.2021.797194)
Supplement: Supplementary file 1 [file Table_1.docx]

**Supplement Table 1 Baseline Characteristics of Study Participants by Two Chemotherapy Protocols Classification**

|  |  | **Chemotherapy Protocols** | | *P* value |
| --- | --- | --- | --- | --- |
| Characteristics | Total | GD-ALL-2008(n=1549) | SCCLG-ALL-2016(n=981) |  |
| Gender, n(%) |  |  |  | 0.052 |
| Male | 1472 (58.2%) | 873 (56.4%) | 599 (61.1%) |  |
| Female | 1058 (41.8%) | 676 (43.6%) | 382 (38.9%) |  |
| Age group(y) |  |  |  | 0.073 |
| ≥1, <10 | 2176 (86.0%) | 1317 (85.0%) | 859 (87.6%) |  |
| ≥10 or <1 | 354 (14.0%) | 232 (15.0%) | 122 (12.4%) |  |
| Initial WBC(×10^9^/L),median(range) | 9.3 (0.1-1095.0) | 9.5 (0.2-1095.0) | 8.9 (0.1-895.5) | 0.355 |
| WBC group, n(%) |  |  |  | 0.102 |
| <10×10^9^/L | 1306 (51.7%) | 796 (51.5%) | 510 (52.0%) |  |
| ≥10×10^9^/L, <50×10^9^/L | 784 (31.0%) | 464 (30.0%) | 320 (32.6%) |  |
| ≥50×10^9^/L | 436 (17.3%) | 285 (18.4%) | 151 (15.4%) |  |
| CNSL, n(%) |  |  |  | 0.394 |
| Yes | 79 (3.1%) | 52 (3.4%) | 27 (2.8%) |  |
| No | 2451 (96.9%) | 1497 (96.6%) | 954 (97.2%) |  |
| ETV6/RUNX1 Status, n(%) |  |  |  | 0.279 |
| Negative | 2390 (94.6%) | 1277 (82.4%) | 792 (80.7%) |  |
| Positive | 137 (5.4%) | 272 (17.6%) | 189 (19.3%) |  |
| Prednisone Response, n(%) |  |  |  | 0.833 |
| PGR | 2314 (92.1%) | 1420 (92.2%) | 894 (92.0%) |  |
| PPR | 198 (7.9%) | 120 (7.8%) | 78 (8.0%) |  |
| D33 BM, n(%) |  |  |  | 0.403 |
| M1 | 2394 (98.1%) | 1468 (98.3%) | 926 (97.8%) |  |
| M2/M3 | 47 (1.9%) | 26 (1.7%) | 21 (2.2%) |  |
| D33 MRD, n(%) |  |  |  | 0.561 |
| <0.01% | 1441 (83.8%) | 1253 (84.0%) | 188 (82.5%) |  |
| ≥0.01% | 279 (16.2%) | 239 (16.0%) | 40 (17.5%) |  |

Abbreviation: WBC, white blood cell;CNSL, central nervous system leukemia; PGR, prednisone good response; PPR, prednisone poor response; MRD, minimal residual disease evaluation; BM, bone marrow.

**Supplement Table 2 Outcome of Study Participants by ETV6/RUNX1 Status Classification**

|  |  | ETV6/RUNX1 status | | *P* value |
| --- | --- | --- | --- | --- |
| Outcome | Total | Negative (n = 2069) | Positive (n = 461) |  |
| Relapse, n(%) | 178 (7%) | 161(7.8%) | 17(3.7%) | <0.001 |
| BM | 130 (5.1%) | 117 (5.7%) | 13 (3%) |  |
| CNS | 30 (1.2%) | 27 (1.3%) | 3 (0.6%) |  |
| Testicle | 13 (0.5%) | 12 (0.57%) | 1 (0.1%) |  |
| Mediastinum | 1 (0.0%) | 1 (0.0%) | 0 (0.0%) |  |
| Others | 4 (0.2%) | 4 (0.19%) | 0 (0.0%) |  |
| Relapse Time (y), median(range) | 1.8 (0.2-5.3) | 1.8 (0.2-5.0) | 2.5 (0.2-5.3) | 0.048 |
